# No contribution of *GSTM1* and *GSTT1* null genotypes to the risk of neutropenia due to benzene exposure in Southeastern Brazil

**DOI:** 10.1590/S1415-47572009005000067

**Published:** 2009-12-01

**Authors:** Carmen Silvia Passos Lima, Gustavo Jacob Lourenço, Irene Lorand-Metze, Helvia Nascimento, Sara Teresinha Ollala Saad, Fernando Ferreira Costa

**Affiliations:** Departamento de Clínica Médica, Faculdade de Ciências Médicas, Universidade Estadual de Campinas, Campinas, SPBrazil

**Keywords:** neutropenia, glutathione S-transferase, *GSTM1*, *GSTT1*

## Abstract

Exposure to benzene has been associated with haematological diseases such as neutropenia (NEB) and acute myeloid leukaemia (AML). We tested whether the null genotypes of the *GSTM1* and *GSTT1* genes, involved in benzene inactivation, altered the risk for NEB in southeastern Brazil. Genomic DNA from 55 NEB patients and 330 controls was analysed by multiplex-polymerase chain reaction. The frequency of the *GSTM1*, *GSTT1* and combined null genotypes was similar in patients and controls (*GSTM1*, 27.3% *vs.* 38.8%, p = 0.16; *GSTT1*, 25.5% *vs.* 19.7%, p = 0.24; *GSTM1*/*GSTT1*, 12.7% *vs.* 6.7%, p = 0.26; respectively). The distribution of genotype classes in NEB patients was similar to normal controls, suggesting that *GSTM1* and *GSTT1* null genotypes make no specific contribution to the risk of NEB. As the *GSTM1* and *GSTT1* null genotypes were previously associated with increased risk for AML in Brazil and elsewhere, we hypothesise that different thresholds of chemical exposure relative to distinct *GSTM1* and *GSTT1* genotypes may determine whether AML or NEB manifests in benzene exposed individuals from southeastern Brazil. Although indicative, our results still require support by prospective and large scale epidemiological studies, with rigorous assessment of daily chemical exposures and control of the possible contribution of other polymorphic genes involved in benzene metabolism.

The past decade has seen an increasing interest in associations of haematological diseases and occupational exposure to chemical agents. In this context, neutropenia due to exposure to benzene (NEB) has generally been characterised by slight to moderate decrease in neutrophil count (Lorand *et al.*, 1984; Ruiz *et al.*, 1994; Queiroz *et al.*, 1997; Augusto *et al.*., 1999). In contrast, a marked reduction in erythrocyte, neutrophil and platelet counts has been seen in acute myeloid leukaemia (AML), a more severe form of occupational disease (Vaughan *et al.*, 2005). Glutathione S-transferases (GST) modulate the effects of exposure to chemical agents linked to NEB and AML (Hayes *et al.*, 2005). The *GSTM1* and *GSTT1* genes have null variant genotypes, which have been associated with AML susceptibility (Ye and Song, 2005).

Occupation-related diseases have been described as a serious health problem in southeastern Brazil (Lorand *et al.*, 1984; Ruiz *et al.*, 1994; Queiroz *et al.*, 1997; Augusto *et al.*, 1999), and associations of the *GST* null genotypes and increased risks for AML were previously reported by us in individuals from this area of the country (Arruda *et al.*., 2001). Moreover, to the best of our knowledge, no studies regarding the associations of the *GSTM1* and *GSTT1* genotypes and NEB risk have been reported. For these reasons, the identification of genotypes of the *GSTM1* and *GST1* genes in NEB patients from southeastern Brazil was considered necessary in order to investigate their influence, if any, as a risk factor for NEB.

We analysed 55 consecutive NEB patients (34 men, 21 women; 29 Caucasians, 26 African-Brazilians; mean age 35 ± 13 years) seen at the hospital of the State University of Campinas, from January 2002 to December 2005. Neutropenia was defined as a neutrophil count below 1.8 x 10^9^/L for Caucasians and 1.5x10^9^/L for African-Brazilians (Watts, 1999; Dale, 2001). NEB patients presented a consistent history of daily benzene occupational exposure (they were painters, mechanics, shoemakers, or workers of construction companies, petrochemical industry and petrol stations) for a period of over 12 months. Hypocellularity of the granulocytic lineage in bone marrow and exclusion of other causes of neutropenia, such as infectious, autoimmune, haematological, thyroid and nutritional deficiency or drug-related neutropenia, as previously recommended by our group (Lima *et al.*, 2006), were also required for NEB diagnosis. The control group consisted of 330 blood donors (247 men, 83 women; 174 Caucasians, 156 African-Brazilians; mean age 51 ± 3 years) without a consistent history of benzene exposure. They were recruited from the same university hospital in order to provide a representative group of the general population that seeks medical assistance in the region. All procedures were carried out according to the principles of the institutional guidelines and all patients and controls provided written informed consent.

Genomic DNA from peripheral blood of patients and controls was analysed by multiplex-polymerase chain reaction for identification of GST genotypes (Arruda *et al.*, 2001). The GST genotypes were analysed after electrophoresis on 2.0% agarose gels (Figure 1), using the β-globin gene as internal control.

Differences between groups were analysed by means of chi-squared or Fishers exact tests. For analysing the associations with NEB, univariate and multivariate analyses were used throughout, in order to obtain odds ratio (OR), adjusted or not for age, gender and ethnic origin, and their corresponding 95% confidence intervals (CI). The statistical package Epi Info was used to perform all these analyses.

The frequencies of the *GSTM1*, *GSTT1* and combined *GSTM1* and *GSTT1* null genotypes were similar in patients and controls. Patients with the distinct genotypes of the *GSTM1* and *GSTT1* genes exhibited similar distribution to normal controls, suggesting that GST genotypes make no significant contribution to NEB, under the chemical exposures encountered in this study (Table 1).

In Brazil, workers are exposed predominantly to solvents, such as benzopyrene, hexachlorobenzene, ethylene oxide, dichloromethane and epoxybutanes, which are metabolised by the GSTM1 and GSTT1 enzymes (Ruiz *et al.*, 1994; Queiroz *et al.*, 1997; Hayes *et al.*, 2005). The chemicals have been consistently associated with both AML and NEB in Brazil (Lorand *et al.*, 1984; Ruiz *et al.*, 1994; Queiroz *et al.*, 1997; Augusto *et al.*, 1999) and in other parts of the world (Cronkite *et al.*, 1989; Ye and Song, 2005; Vaughan *et al.*, 2005). These data supported the association of both *GST* null genotypes with increased risks for AML previously found by our group (Arruda *et al.*, 2001). On this basis, the *GST* null genotypes were also expected to be associated with increased NEB risk. Surprisingly we found similar frequencies of the *GST* genotypes in our NEB patients and controls.

Unfortunately, there was no available data concerning the levels of benzene exposure of the NEB patients in this study and of the AML patients in our previous study (Arruda *et al.*, 2001). We assumed similar exposures to benzene for patients in either group. Taking these results together, we hypothesise that different thresholds of chemical exposure relative to distinct GST genotypes may determine whether NEB or AML arises in chemical hazard exposed individuals from southeastern Brazil. Thus, those highly exposed to chemicals and homozygous for the null GST alleles may develop AML, since this seems to be more dependent on the GST pathway of carcinogen metabolism, whilst those individuals with less exposition to chemicals may be less dependent on carcinogen inactivation by the GST isoenzymes, and therefore more prone to present the benign form of occupational disease, NEB, without mediation of GST genotypes. Although indicative, these results must, however, be confirmed by prospective studies with larger samples of NEB and AML patients and controls, with rigorous assessment of daily chemical exposures, and control of the influence of other polymorphic genes involved in benzene metabolism (Aydin-Sayitoglu *et al.*, 2006).

**Figure 1:**
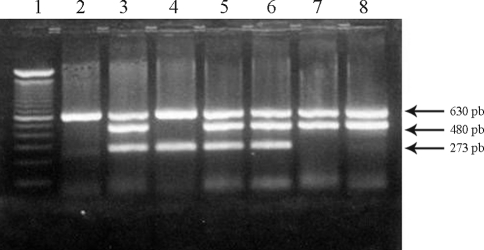
Multiplex-PCR for detection of the wild and null alleles of the glutathione S-transferase mu1 (*GSTM1*) and theta1 (*GSTT1*) genes. Ethidium bromide-stained 2% agarose gel showing products of 273 bp and 480 bp corresponding to the *GSTM1* and *GSTT1* genes, respectively. The 630 bp product corresponds to a β-globin gene fragment, the internal control. Lane 1, 100 bp DNA ladder. Lane 2, individual with combined *GSTM1* and *GSTT1* null genotypes. Lanes 3, 5 and 6, individuals with both *GSTM1* and *GSTT1* genes. Lane 4, individual homozygous for the *GSTT1* null allele. Lanes 7 and 8, individuals homozygous for the *GSTM1* null allele.

## Figures and Tables

**Table 1:** *GSTM1* and *GSTT1* genotypes in 55 patients with neutropenia due to exposure to benzene and 330 controls.

	*GSTM*			*GSTT1*			*GSTM1*/*GSTT1*		
	Present n (%)	Null n (%)		Present n (%)	Null n (%)		Both present n (%)	One null n (%)	Both null n (%)
Cases	40 (72.7)	15 (27.3)		41 (74.5)	14 (25.5)		33 (60.0)	22 (40.0)	7 (12.7)
Controls	202 (61.2)	128 (38.8)		265 (80.3)	65 (19.7)		159 (48.2)	149 (45.1)	22 (6.7)
OR (CI 95%)	1.0 (ref)	0.59 (0.31-1.11)		1.0 (ref)	1.39 (0.72-2.71)		1.0 (ref)	0.71 (0.40-1.28)	1.53 (0.60-3.88)
*P value*	0.13			0.37				0.30	0.43
OR* (CI 95%)	1.0 (ref)	0.61 (0.31-1.21)		1.0 (ref)	1.54 (0.75-3.14)		1.0 (ref)	0.74 (0.39-1.38)	0.82 (0.65-4.81)
*P* value	0.16			0.24				0.34	0.26

n: number of cases; OR: odds ratio; *: adjusted OR by age, gender, and ethnic origin; CI: confidence interval.

## References

[Arrudaetal2001] Arruda V.R., Lima C.S., Grignoli C.R., Melo M.B., Lorand-Metze I., Alberto F.L., Saad S.T.O., Costa F.F. (2001). Increased risk for acute myeloid leukaemia in individuals with glutathione S-transferase mu 1 (GSTM1) and theta 1 (GSTT1) gene defects. Eur J Haematol.

[Augustoetal1999] Augusto L.G., Fontbonne A., De Carvalho E.M., Novaes E.M., Novaes T.C. (1999). Socio-medical intervention in occupational health: Benzenism in Brazil. Int J Occup Environ Health.

[Aydin-Sayitogluetal2006] Aydin-Sayitoglu M., Hatirnaz O., Erensoy N., Ozbek U. (2006). Role of the *CYP2D6*, *CYP1A1*, *CYP2E1*, *GSTT1*, and *GSTM1* genes in the susceptibility to acute leukemias. Am J Hematol.

[Cronkiteetal1989] Cronkite E.P., Drew R.T., Inoue T., Hirabayashi Y., Bullis J.E. (1989). Hematotoxicity and carcinogenicity of inhaled benzene. Environ Health Perspect.

[Dale2001] Dale D.C., Beutler E., Lichtman M.A., Coller B.S., Kipps T.J. (2001). Neutropenia and Neutrophilia. Hematology.

[Hayesetal2005] Hayes J.D., Flanagan J.U., Jowsey I.R. (2005). Glutathione transferases. Annu Rev Pharmacol Toxicol.

[Limaetal2006] Lima C.S.P., Paula E.V., Takahashi T., Saad S.T.O., Lorand-Metze I., Costa F.F. (2006). Causes of incidental neutropenia in adult patients. Ann Hematol.

[Lorandetal1984] Lorand I.G.H., Souza C.A., Costa F.F. (1984). Haematological toxicity associated with agricultural chemicals in Brazil. Lancet.

[Queirozetal1997] Queiroz M.L.S., Bincoleto C., Perlingeiro R.C.R., Souza C.A., Toledo H. (1997). Defective neutrophil function in workers occupationally exposed to hexachlorobenzene. Hum Exp Toxicol.

[Ruizetal1994] Ruiz M.A., Augusto L.G.S., Vassallo J., Vigorito A.C., Lorand-Metze I., Souza C.A. (1994). Bone marrow morphology in patients with neutropenia due to chronic exposure to organic solvents. Pathol Res Pract.

[Vaughanetal2005] Vaughan A.T., Betti C.J., Villalobos M.J., Premkumar K., Cline E., Jiang Q., Diaz M.O. (2005). Surviving apoptosis: A possible mechanism of benzene-induced leukemia. Chem Biol Interact.

[YeandSong2005] Ye Z., Song H. (2005). Glutathione S-transferase polymorphisms (GSTM1, GSTP1 and GSTT1) and the risk of acute leukaemia: A systematic review and meta-analysis. Eur J Cancer.

[Watts1999] Watts R.G., Lee G.R., Foerster J., Lukens J., Paraskevas F., Greer J.P., Rodgers G.M., Wintrobe M.M. (1999). Neutropenia. Wintrobe's Clinical Hematology.

